# Chemical analysis of essential oil extracted from pomelo sourced from Bangladesh

**DOI:** 10.1016/j.heliyon.2022.e11843

**Published:** 2022-11-30

**Authors:** Shohag Chandra Das, Mobarak Hossain, Mohammad Zakaria Hossain, Nusrat Jahan, Mohammad Abbas Uddin

**Affiliations:** a Department of Wet Process Engineering, Bangladesh University of Textiles, Dhaka, Bangladesh; b Department of Industrial and Production Engineering, Bangladesh University of Textiles, Dhaka, Bangladesh; c Department of Dyes and Chemical Engineering, Bangladesh University of Textiles, Dhaka, Bangladesh

## Abstract

This research aims to extract essential oils from the peel of two varieties of *Citrus maxima*- White pomelo and Red pomelo of Bangladeshi origin by hydrodistillation (HD) method and characterization of the extracted oils. The study also looked into the effect of different conditions, such as type of peels, and extraction time, on the yield amount. To determine the chemical components of oil, the Gas Chromatography-mass spectrum (GC-MS) technique has been used. The three major components were, for *white pomelo,* limonene (67.58%), β-linalool (7.53%) and Neral (6.61%), and for *red pomelo*, limonene (73.82%), β-linalool (5.42%) and Neral (4.11%). The morphological changes in the oil glands of the peels of both varieties were compared to understand changes before and after the extraction. The result showed that white pomelo (WP) provides a slightly higher yield percentage in similar extraction time than red pomelo (RP), 1.09 and 0.96%, respectively. GC-MS results showed that the presence of limonene is the highest for both pomelos, although the amount is higher in RP than that of WP. However, the digital microscopy showed the drawbacks of the hydrodistillation process. The pressure in the oil glands during the distillation is too low to rupture the oil glands fully. This study will be able to broaden the path for future studies on related physicochemical and biochemical properties of pomelo varieties of Bangladesh.

## Graphical abstract

**Figure:**
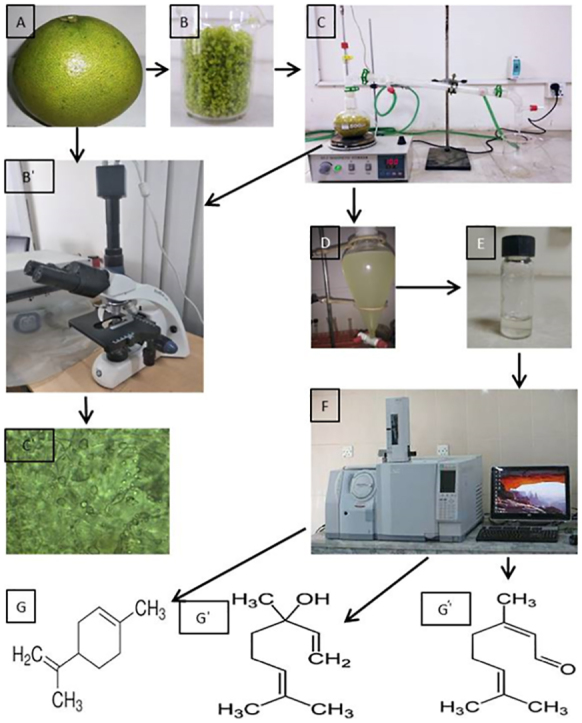

## 1 Introduction

From ancient times, essential oils were extracted from odorous plants or different resinous parts of plants in India, Persia, and Egypt Naeem et al. 2018). The oil is usually hydrophobic and is composed of other volatile compounds, such as acid, alcohols, aldehydes, ketones, esters, and hydrocarbons. Due to no reasonable side effects, natural essential oils have been more popular in commercial use (Shakir and Salih 2015; Ranitha et al. 2014; Arce and Soto 2008). The small oil glands of the peel contain the oil made up of different components and determine the final characteristics of the oil (Ferhat et al. 2006). However, The components of the essential oil from natural sources depend on various factors, such as soil (nutrients, fertiliser, biotic compounds), origin, geography, climate (temperature, winds, rainfall, altitude, latitude, insolation), ripeness, post-treatment, extraction method (Arce and Soto 2008).

Pomelo is a citrus food of the *Rutaceae* family, which is the largest among all citrus families in size (Chen et al. 2016). Its scientific names are *Citrus maxima* or *Citrus grandis* with other varieties such as *Citrus sinensis L.*, *Citrus latifolia,* and *Citrus paradisi. L.* (Mabberley 1997)*.* It is mostly found in Southeast Asia, containing, on average 89% of water, 10% of carbohydrates, 1% of protein with little fat (Lan-Phi and Vy 2015), and vitamin C like other citrus fruits (Nielsen 2017). The most noticeable factor is its antioxidant, anti-melanogenic, anti-hypertensive, and anticoagulant properties (He et al. 2019; Okoh et al. 2010). The physicochemical properties of extracted essential oil of pomelo peels were studied intensively in Hong Kong (Chen et al. 2016), Vietnam (Hien et al. 2018), (Lan-Phi and Vy 2015), China (He et al. 2019), Kenya (Njoroge et al. 2005), Turkey (Uysal et al. 2011), Brazil (Atti-santos et al., 2005) and Algeria (Ferhat et al., 2006). However, pomelo variants from Bangladeshi sources were not studied and were not within the public domain. As the properties of any natural fruits can be varied with the variation of the region (Islam et al. 2013; Kumar and Tripathi 2011), therefore, this study addressed the two most available varieties of pomelo in Bangladesh - white pomelo (WP) and red pomelo (RP).

There are various extraction techniques for essential oil, such as hydrodistillation (HD), cold expression, steam distillation, solvent extraction, maceration, empyreumatic (or destructive) distillation (Tripathi 2011). Hydrodistillation is the most commonly used and quick and easy to apply (Ranitha et al., 2014; Stratakos and Koidis 2016); however, the deterioration of the oil extracted in the hydrodistillation method is higher due to the instability of components after extraction (Atti-santos et al., 2005). He et al. (2019) extracted the pomelo peel essential oil by cold pressing method and determined chemical composition by gas chromatography-mass spectrometry (GC/MS), measured antioxidant and anti-melanogenic properties of the oil. Microwave-assisted hydrodistillation and surface response methodology were used to extract pomelo essential oil in Vietnam (Hien et al. 2018). Lan-Phi and Vy (2015) investigated chemical compositions and their antioxidant and antibacterial activities in essential oils extracted from different varieties of pomelo peel grown in the South of Vietnam. Dao et al. (2022) extracted essential oils from pomelo (*Citrus maxima*) peel by HD from Ben Tre Province, Vietnam, where lemonine was the main component, and pseudo first order and pseudo-second-order kinetic models in both linear and nonlinear forms were also derived. The essential oil from the peels of Mandarin (Tien Giang province, Vietnam) were extracted by HD, Microwave-assisted hydrodistillation (MAHD), and Microweave extraction (ME) technique and the result in GC-MS showed that limonene was the highest, followed by - Sabinene as the lowest present components among total five components (Dao et al. 2020). Essential oils extracted by MAHD method from orange leaves (Vietnam) and a statistical model through analysis of variance (ANOVA) were established for the highest essential oil yield, where sabinene (30.556 %), Cis-ocimene (10.139 %), and D-limonene (9.682 %) as major components (Dao et al., 2019).

Chen et al. (2016) used the solvent-free microwave method to extract essential oils from pomelo peel and the hot-solvent microwave extraction method to extract pectin. The essential oils of redblush (*Citrus paradisi Macfadyen forma Redblush)* and pomelo (*Citrus grandis Osbeck*) varieties were extracted by cold-pressed method from Kenya, and components were determined by GC and GC-MS (Njoroge et al. 2005). The essential oil of pomelo from Turkey origin was extracted by solvent-free microwave extraction and hydrodistillation – and their chemical composition and antibacterial performance were analysed (Uysal et al. 2011).

In this study, the essential oil was extracted from two variants of pomelo from Bangladeshi origin using the hydrodistillation method. The properties that were compared are yield percentages, chemical composition, and morphological changes of the oil glands to find the difference in properties and compare with other pomelo varieties of different regions keeping the processes the same.

## 2 Material and methods

### 2.1 Materials

**Figure 1:**
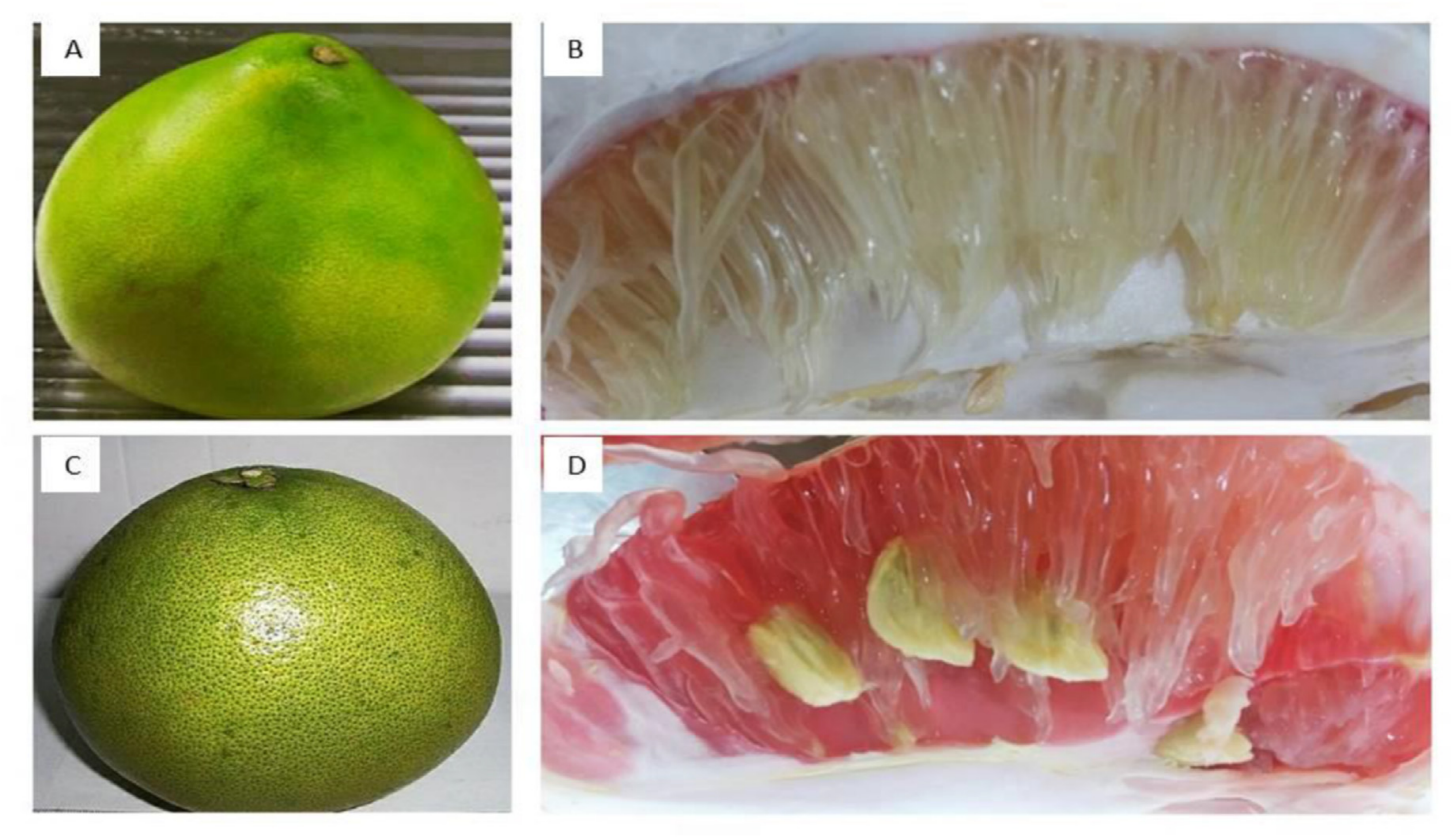
Pomelo fruits collected from Dhaka city, A) whole white pomelo, B) inside cells of white pomelo; C) whole red pomelo; D) inside cells of red pomelo.

Raw pomelos*, Citrus maxima* (WP, RP) were collected from the local markets of Dhaka sourced in Chittagong hill tricks (21°33′ north latitude and 92°24′ east longitude). The fresh and mature fruits were collected, and the fruits were washed using distilled water and dried at room temperature. There is a little variation in the outside appearance of the variant – WP, Figure 1(A), is larger than RP, Figure 1(C) with a thicker peel and harder skin. The cells inside pomelo fruits are red and white, as seen in Figure 1 (B) and Figure 1(D).

### 2.2 Hydrodistillation extraction of essential oil

**Figure 2:**
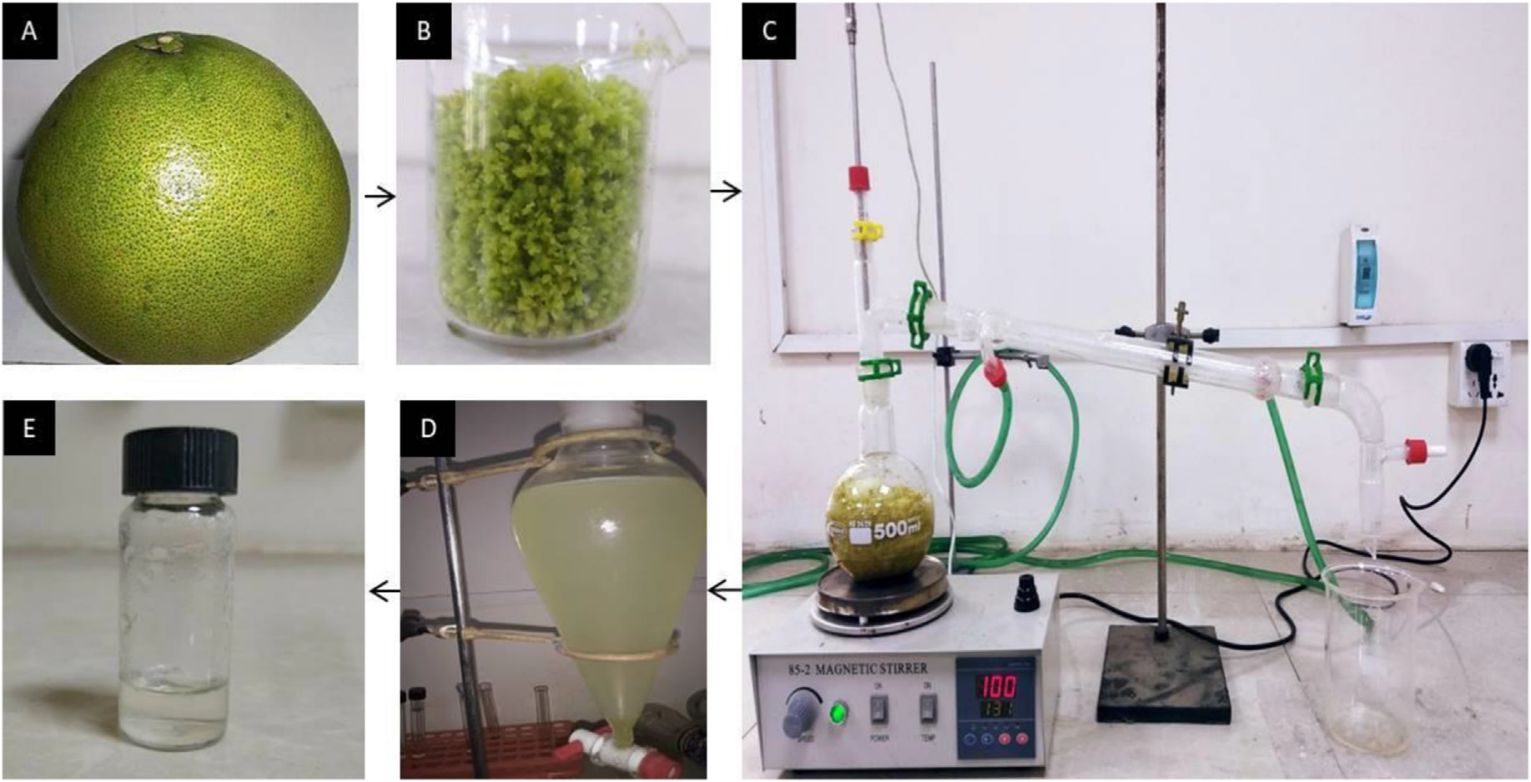
Schematic diagram of the study; A) whole pomelo; B) grinded peel; C) Hydrodistillation setup; D) Separator funnel E- Extracted oil.

At first, the peels were separated from the pomelo, Figure 2(A), which was cut into about small pieces (20 mm × 20 mm x 2mm), giving a yield of approximately 20% (w/w) of peels compared to the whole fruit. Then the peels were weighed and then grinded with a grinder to extract the oil efficiently, Figure 2(B). The grinded peels were added with distilled water (300 ± 5 g) in a volumetric flux with a ratio of about 1:3 (peels:water) and mixed properly. The mixture was set up in a distillation chamber on a magnetic stirrer heater and stirred continuously. This solid-liquid mixture was heated up to 100 ± 2 °C, and steam helped release the essential oil from the peels. The volatile oil and vapour mixture passed through the cold pipe, which got the heat exchange from the continuous cooling water flow, making the condensed oil-water mixture stored in a beaker, Figure 2(C). The extracted oil-water mixture contained the essential oil floating on the water due to their density difference. After that, the mixture was transferred into a separator funnel, Figure 2(D), and kept 24 h for the separation (Lan-Phi and Vy 2015). Then the desired oil was separated, Figure 2(E), from the mixture by the separator funnel. Initially, all extractions were carried out several times to establish the methods, and then the experiments were conducted at least three times and their average value were taken. and related stastical analysis had been conducted with Burette.

**Figure 3:**
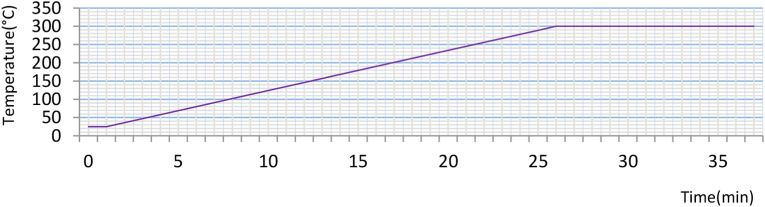
The thermal cycle of gas chromatography (same for both varieties).

### 2.3 Yield percentage

The following Eq. (1) is used to evaluate the extraction yield percentage.

(1) $$Yield\;\%=\frac{amount\;of\;oil\;extracted\;\left(gm\right)*100\%}{total\;amount\;of\;peel\;\left(gm\right)}$$

### 2.4 Chemical composition analysis

The Gas Chromatography-Mass spectrometry (model GCMS-QP2010 SE, Japan) was conducted at the Bangladesh Council of Scientific and Industrial Research (BCSIR) lab to determine the chemical composition of both essential oils-with a turbomolecular pump (58 L/s for He) and a rotary pump 30 L/min (60Hz) equipped with Rxi-624silMS capillary column (length 30m, diameter 0.32 mm and thickness 1.80μm). The GC analysis was conducted in the following conditions: helium carrier gas with flow rate 1.40 ml/min; split ratio of 50.0; injection volume 1.0 μl; injection temperature 250 °C; Oven temperature 40 °C (40 °C to 300 °C in 27 min), sampling time 1 min; injection mode splitless; ionisation temperature 200 °C, raw control mode pressure (9.7 kPa); interface temperature 250 °C and solvent cut time 6 min. Then the components were determined. Two repeat experiments were conducted for each type for validation of the result. The relative concentration of the compounds was taken from the GC-MS by matching with prestored data of the software (see ​Figure 3).

### 2.5 Morphological analysis

The morphology of the treated peel was compared with untreated peel by digital microscopy at the Center of Advanced Research in Science (CARS), Dhaka. Both the varieties were observed on the surface and cross-sectional at 40 times magnification. The specimens were sliced into very thin layers to observe on the microscope (Euromex BioBlue 4260, Netherland).

## 3 Result and discussion

### 3.1 Extraction yield

The percentage yield of WP and RP is 1.09 ± 0.007% and 0.96 ± 0.0085%, respectively (WP is slightly higher than RP - around 0.13%). Although the difference is insignificant, this is consistent with other studies (Lan-Phi and Vy 2015) proving that the pomelo variety and growing location can affect yield%. Moreover, the extraction procedure also plays an important role, and optimizing the procedure is vital (Ranitha et al. 2014).

**Figure 4:**
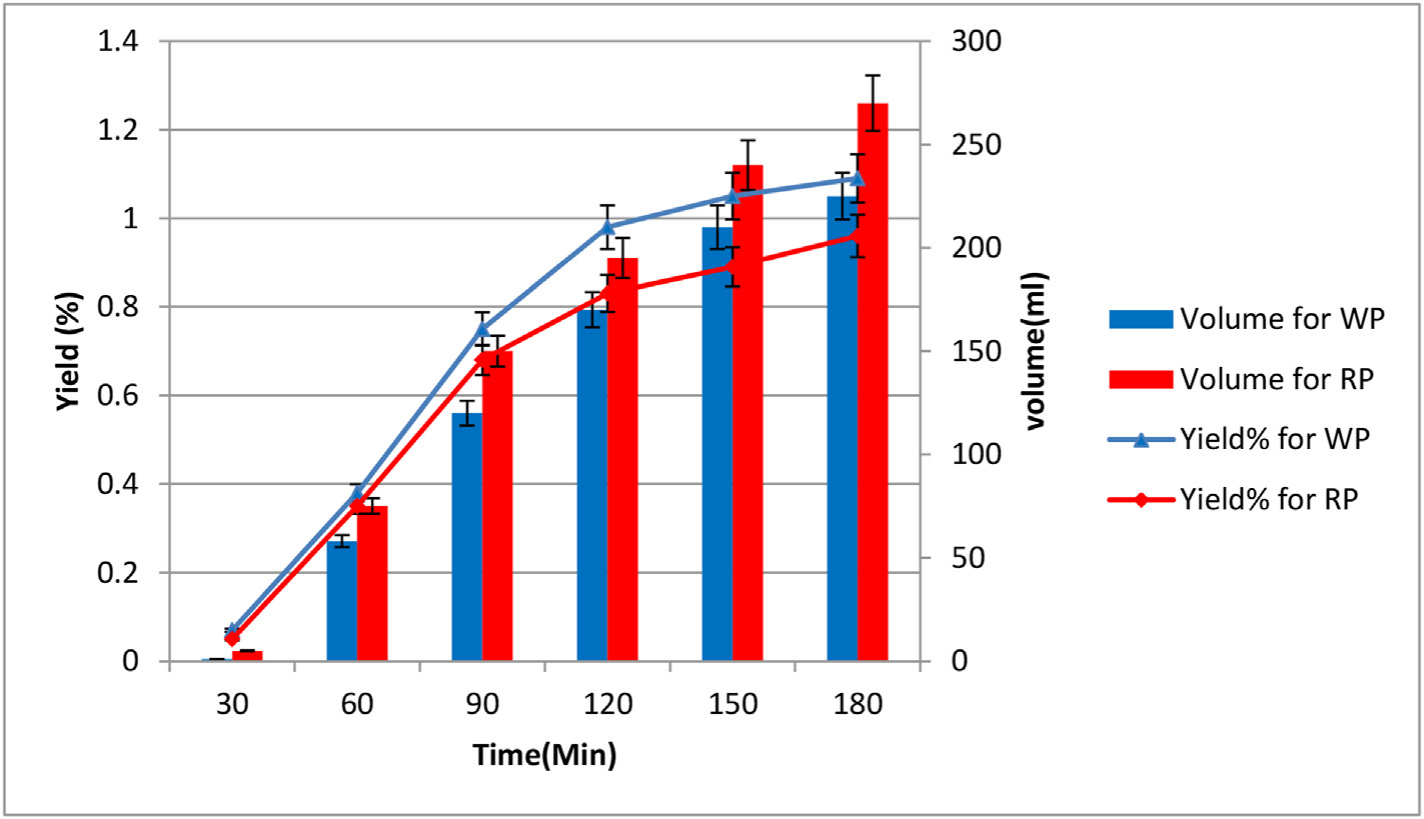
Effect of time on oil Yield % (primary axis vs horizontal axis) and effect of time on water-oil mixture amount (Secondary axis Vs horizontal axis).

The time vs yield study in Figure 4 shows that the extraction amount has increased with time. However, the extraction rate for the first 2 h was higher than the end as the amount of oil diminished over time as most of the oils from the oil gland had come out with steam from the first 30 min–120 min for both varieties. Figure 4 also shows the volume of extracted oil-water mixture after hydrodistillation – 225 mL for WP variety and 270 mL for RP variety after 3 h. However, the oil is lower in the latter case.

**Figure 5:**
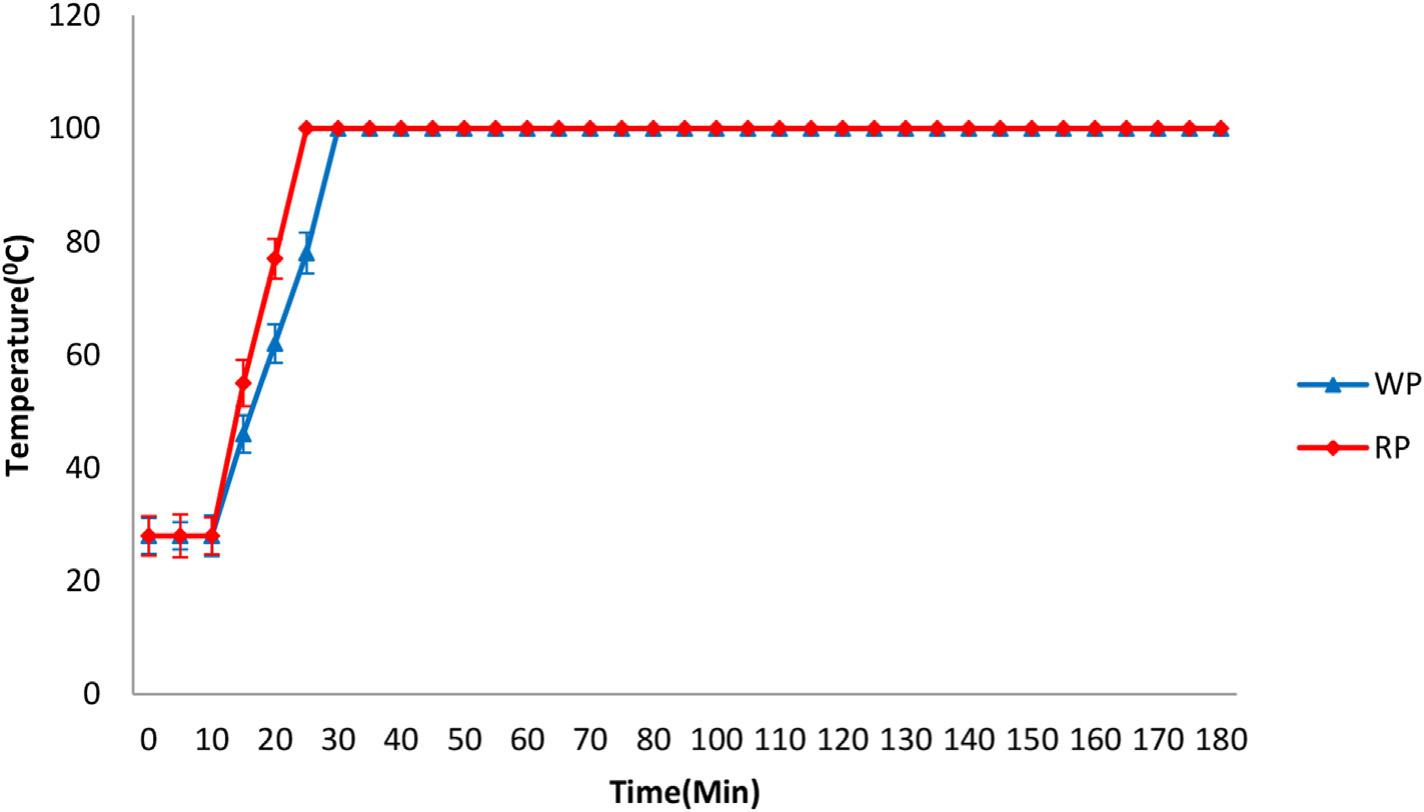
Change in mixture temperature with time.

The time vs. temperature graph in Figure 5 showed that to reach 100 °C, on average, the WP variety took 31 min, and the RP variety took 27 min due to the ingredients in the mixture that can affect the boiling temperature of a mix (Thome 1989). After the first 10 min, the temperature increased from room temperature (28 °C) for both varieties. The temperature at the first drop to the highest temperature for the oil-water mixture was a few seconds in the case of both types. These factors can be explained by the fact that the ingredients in the mix can affect the boiling temperature.

### 3.2 Morphological analysis

**Figure 6:**
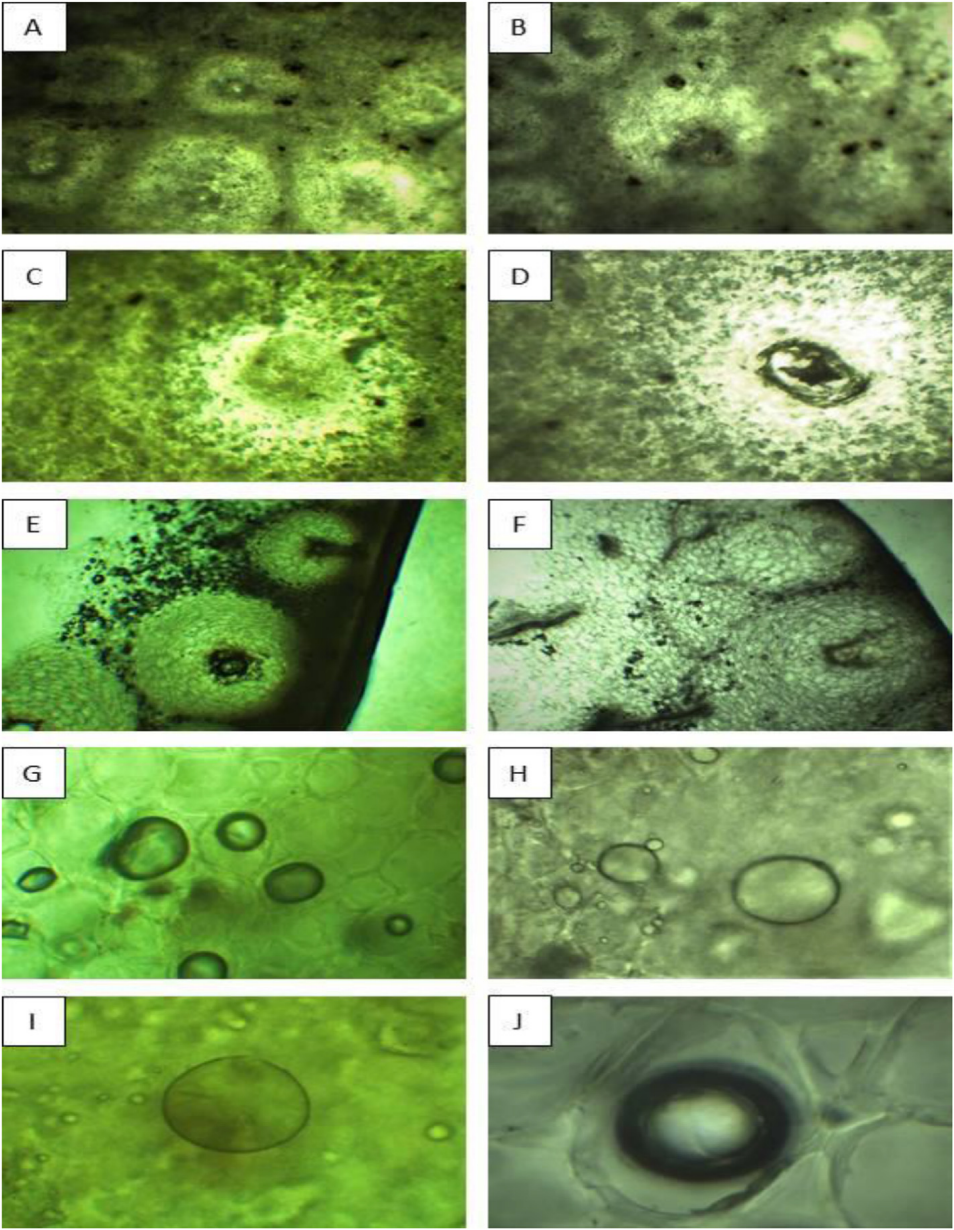
*Digital microscopy of pomelo peels (A-J×40); Untreated sample (A-F); treated sample (G-J); WP (A, C, E, G, I); RP (B, D, F, H, J)*.

Figure 6 shows the top and cross-sectional view of the peel of both varieties before and after extraction at 100 °C for 3 h Figures 6(A) and Figure 6(B) show the oil glands of WP and RP, respectively, at 40 times magnification. Figures 6(C) and Figure 6(D) show only a single gland at the same magnification. This support that the gland of WP is slightly larger than RP, which could be the possible reason for the variation in oil yield percentage. Figures 6(E) and Figure 6(F) are the cross-sections of the peel where the oil glands stay in the periphery. Figures 6(G) and Figure 6(H) show that the treated glands have shrunken compared to before, but there are still oil sacks. Both varieties contain the oil, but the RP has a comparatively larger amount left than WP. Figures 6(I) and Figure 6(J) view a single gland and show the presence of undamaged glands indicating hydrodistillation cannot exert enough pressure to rupture the glands fully, which is a drawback of the method. Nevertheless, it is one of the reliable and easy processes for essential oil extraction. The finding shows that WP possesses more oil than RP, and the oil amount in the after-treatment gland is higher in RP than in WP.

### 3.3 Chemical composition analysis

Table 1 shows the volatile compounds in the oil extracted from the pomelo varieties. There were eighteen main components that were detected in WP and RP, with a total amount of 97.7% and 98.75%, respectively. The main component in WP and RP was limonene (a hydrocarbon), with relative concentrations of 67.58% and 73.82%. However, the second, third and fourth most amounts were β-linalool (Alcohol), Neral (Aldehyde), and Nootkatone (Ketone), where WP and RP contained 7.53% and 5.42% β-linalool, 6.61%, and 4.11% Neral and 4.98% and 1.78% Nootkatone. On the other hand, α-Pinene (Hydrocarbon), Terpene-4-ol (Alcohol), β-Citral (Aldehyde), and Germacrene D (Ketone) were higher in RP than WP with 1.45% and 1.67% α-Pinene, 0.20% and 1.47% Terpene-4-ol, 0.4% and 0.67% β-Citral and 0.45% and 0.52% Germacrene D. However, β-Myrcene which was a major component of WP (3.49%) but absent in RP. In the same way, Ocimene (1.67%), Citronellal (1.01%), Caryophyllene (0.39%), and Cis-α-Bisabolene (0.49 %) were also present in WP but absent in RP. On the other hand, three compounds (Mesitylene-3.34%, β-Pinene - 2.55%, and Durene-2.97%) were present in RP but absent in WP. It was also reported that the major component in pomelo was the limonene and citrus family to produce fragrance (He et al. 2019; Lan-Phi and Vy 2015).

**Table 1:** Chemical composition (%) of the extracted oil from WP and RP varieties extracted by Hydrodistillation method.

No	Compounds	Chemical Group	Molecular Formula	Boiling temp (°C)	Retention time (min)	WP conc. %	RP conc. %
1	α-Pinene	Hydrocarbon	C_10_H_16_	155	7.67	1.45	1.67
2	Mesitylene	Hydrocarbon	C_10_H_12_	164.7	7.99	---	3.34
3	β-Pinene	Hydrocarbon	C_10_H_16_	165	8.12	---	2.55
4	β-Myrcene	Hydrocarbon	C_10_H_16_	167	8.73	3.49	---
5	Limonene	Hydrocarbon	C_10_H_16_6	176	9.763	67.58	73.82
6	Ocimene	Hydrocarbon	C_10_H_16_	100	9.81	1.67	---
7	Durene	Hydrocarbon	C_10_H_14_	192	10.25	---	2.97
8	β-linalool	Alcohol	C_10_H_18_O	198	10.58	7.53	5.42
9	Citronellal	Ketone	C_10_H_18_O	208	11.38	1.01	---
10	Terpene-4-ol	Alcohol	C_10_H_18_O	211	12.47	1.20	1.47
11	Neral	Aldehyde	C_10_H_16_O	225	12.85	6.61	4.11
12	β-Citral	Aldehyde	C_10_H_16_O	227	13.28	0.4	0.67
13	α-Citral	Aldehyde	C_10_H_16_O	228	13.63	0.07	0.2
14	Caryophyllene	Hydrocarbon	C_15_H_24_	262	15.34	0.39	---
15	Cis-α-Bisabolene	Hydrocarbon	C_15_H_24_	276	16.123	0.49	---
16	Germacrene D	Ketone	C_15_H_24_	236.4	16.47	0.45	0.52
17	β- Bisabolene	Hydrocarbon	C_15_H_24_	274	16.67	0.38	0.23
18	Nootkatone	Ketone	C_15_H_22_O	170	21.55	4.98	1.78
					Total	97.7	98.75

## 4 Discussion

The study of (He et al. 2019) showed that the major component was limonene (55.92%), followed by β-myrcene (31.17%) and β-pinene (3.16%), and in this study, major components were also found to be limonene, β-linalool, and Neral (Lan-Phi and Vy 2015). Also, limonene is the major component of all five varieties in south Vietnam, but the highest amount of limonene was 95.7% for DC-DN variety, and the lowest was 67.2% for NR-VL variety. The finding also supported our finding that the components present in one variable can be absent in other. Chen et al. (2016) also found limonene as the highest amount (82.58%), with the total amount of all major components 95.58% in hydrodistillation method. In contrast, this study resulted in 97.7% and 98.75% for WP and RP. However, the authors Chen et al. (2016) used 34, whereas this current study found 18 major components. In Kenya, total hydrocarbons were found to be 93.3% for red blush and 97.5% for pomelo (Njoroge et al., 2005), whereas in this study, total hydrocarbons were found to be 75.45% for WP and 84.58% for RP. Moreover, alcohol was 1.4% for red blush and 0.3% for pomelo, and we got 8.73% for WP and 6.89% for RP. Table 2 shows the relative comparison of different varieties of pomelo.

**Table 2:** Major three components with the percentage of different variety of pomelos in different countries.

Pomelo Variety	Extraction Method	Major three components		Source	Reference
DX-BT	Cold Press	Limonene	69.4%	Da Xanh in Ben Tre province, Vietnam	(Lan-Phi and Vy 2015)
		β-phellandrene	12.8%		
		Myrcene	8.6%		
NR-VL	Cold Press	Limonene	67.2%	Nam Roi in Vinh Long province, Vietnam	(Lan-Phi and Vy 2015)
		γ-terpinene	9.9%		
		β-phellandrene	9.2%		
BL-DT	Cold Press	Limonene	77.6%	Buoi Long in Dong Thap province, Vietnam	(Lan-Phi and Vy 2015)
		γ-terpinene	13.5%		
		α-pinene	2.2%		
DX-DT	Cold Press	Limonene	95.7%	Duong Cam in Dong Nai province, Vietnam	(Lan-Phi and Vy 2015)
		Myrcene	1.9%		
		α-pinene	0.6%		
*Citrus sinensis* Osbeck	Hydrodistillation	Limonene	77.4%	Iran	(Golmohammadi, Borghei, and Zenouzi, 2018)
		β-Myrcene	6.1%		
		L-Linalool	5.1%		
Shatin pomelos (Teaka)	SFME at 450 W	Limonene	80.7%	Hong Kong	(Chen et al., 2016)
		Nootkatone	8.9%		
		β-Myrcene	3.7%		
Shatin pomelos (Teaka)	SFME at 300 W	Limonene	86.5%	Hong Kong	(Chen et al., 2016)
		Nootkatone	3.9%		
		β-Myrcene	2.1%		
Shatin pomelos (Teaka)	SFME at 150 W	Limonene	78.1%	Hong Kong	(Chen et al., 2016)
		Nootkatone	9.3%		
		β-Myrcene	1.5%		
Shatin pomelos (Teaka)	Hydrodistillation	Limonene	82.6%	Hong Kong	(Chen et al., 2016)
		Nootkatone	4.8%		
		β-Myrcene	2.5%		
Redblush	Cold Press	Limonene	91.1%	Kenya	(Njoroge et al., 2005)
		α-pinene	0.5%		
		sabinene and (Z)-carvone	0.4%		
Pummelo	Hydrodistillation	Limonene	94.8%	Kenya	(Njoroge et al., 2005)
		α-pinene	0.5%		
		sabinene	0.4%		
China pomelo	Cold Press	Limonene	55.9%	Guan Xi, China	(He et al., 2019)
		β-Myrcene	31.2%		
		α-pinene	3.2%		
*Citrus Paradisi. L*	SFME	Limonene	91.5%	Turkey	(Uysal et al., 2011)
		trans-Limonene oxidec	0.9%		
		β-pinene	0.8%		
*Citrus Paradisi. L*	Hydrodistillation	Limonene	88.6%	Turkey	(Uysal et al., 2011)
		β-pinene	1.2%		
		α-Terpinene	1.0%		
*Citrus latifolia (Tanaka)*	Hydrodistillation	d-limonene	47.5%	Brazil	(Atti-santos et al., 2005)
		β-pinene	12.4%		
		γ-Terpinene	12.3%		
*Citrus latifolia (Tanaka)*	Supercritical extraction	d-limonene	48.9%	Brazil	(Atti-santos et al., 2005)
		γ-Terpinene	17.0%		
		β-pinene	14.5%		
*Citrus sinensis L.*	MAD	Limonene	76.7%	Algier, Algeria	(Ferhat et al., 2006)
		β-Myrcene	4.3%		
		Linalool	3.1%		
*Citrus sinensis L.*	Hydrodistillation	Limonene	78.5%	Algier, Algeria	(Ferhat et al., 2006)
		β-Myrcene	5.3%		
		β-pinene	2.7%		
*Citrus maxima* (white pomelo)	Hydrodistillation	Limonene	67.58%	Dhaka, Bangladesh	This study
		β-linalool	7.53%		
		Neral	6.61%		
*Citrus maxima* Red Pomelo)	Hydrodistillation	Limonene	73.82%	Dhaka, Bangladesh	This study
		β-linalool	5.42%		
		Neral	4.11%		

These findings support that the chemical composition difference in essential oil depends on various factors (Olfa et al. 2021). In addition, the physicochemical and biological properties like antimicrobial activity, antioxidant property, flavour, boiling temperature depend on the chemical composition. The component β- Bisabolene has anticancer properties (Jou et al. 2016).

Among all referred studies, Ferhat et al. (2006) for *Citrus sinensis L.* by MAD method was much closer to this recent finding - the three major components were limonene (76.7%), β-myrcene (4.3%), and linalool (3.1%). On the other hand, in this study, the three major components were, for *Citrus maxima*, limonene (67.58%), β-linalool (7.53%) and Neral (6.61%), and for *Citrus grandis*, limonene (73.82%), β-linalool (5.42%) and Neral (4.11%). We can conclude that these subtle differences could be due to various factors, such as the geographical position, a climatic conditions such as temperature, winds, rainfall, and soil quality such as nutrients, fertilisers, and biotic compounds, along with various extraction techniques.

## 5 Conclusion

This study extracted essential oils from the peel of two varieties of pomelo of Bangladesh by hydrodistillation method. The chemical composition of the oils and morphological change of the peel after treatment has been evaluated. We found that white pomelo (WP) gave a little more extraction yield than red pomelo (RP). The chemical compositions showed that the compounds in the oil were affected by the pomelo varieties. The main components were limonene, β-linalool, Neral, β-Myrcene, and Nootkatone. The morphological changes, viewed by digital microscopy, of both varieties after treatment compared with the before treatment showed the changes in the oil glands, which also vary with the pomelo types. After treatment, the condition of the oil glands determined the extraction yield % and other properties. Further research should be conducted regarding other physicochemical and biochemical properties of pomelo varieties, such as antimicrobial, antibacterial, and antioxidant properties.

## Declarations

### Author contribution statement

Shohag Chandra Das, BSC; Mobarak Hossain, BSC; Mohammad Zakaria Hossain, BSC; Nusrat Jahan, MSC; Mohammad Abbas Uddin, PhD: Conceived and designed the experiments; Performed the experiments; Analyzed and interpreted the data; Contributed reagents, materials, analysis tools or data; Wrote the paper.

### Funding statement

Dr Mohammad Abbas Uddin was supported by Bangladesh University Of Textiles internal fund [BUTEX/2019/RnE/002].

### Data availability statement

Data included in article/supp. material/referenced in article.

### Declaration of interests statement

The authors declare no conflict of interest.

### Additional information

No additional information is available for this paper.
